# Supplementary material for: Significant contribution of small icebergs to the freshwater budget in Greenland fjords
Source: Commun Earth Environ. 2020 Oct 15;1(1):31. doi: 10.1038/s43247-020-00032-3 (PMC7594622; doi:10.1038/s43247-020-00032-3)
Supplement: Supplementary file 1 — Supplementary Information [file 43247_2020_32_MOESM1_ESM.pdf]

Supplementary Information for
"Significant contribution of small icebergs to the freshwater budget in Greenland fjords"

Soroush Rezvanbehbahani[1,2], Leigh A. Stearns[1,2], Ramtin Keramati[3],
Siddharth Shankar[1,2], C.J. van der Veen[4]

[1]Department of Geology, University of Kansas, Lawrence, KS 66045

[2]Center for Remote Sensing of Ice Sheets, University of Kansas, Lawrence, KS 66045

[3]Institute of Computational and Mathematical Engineering, Stanford University, Stanford, CA 94305

[4]Department of Geography and Atmospheric Science, University of Kansas, Lawrence, KS 66045

Supplementary Figures

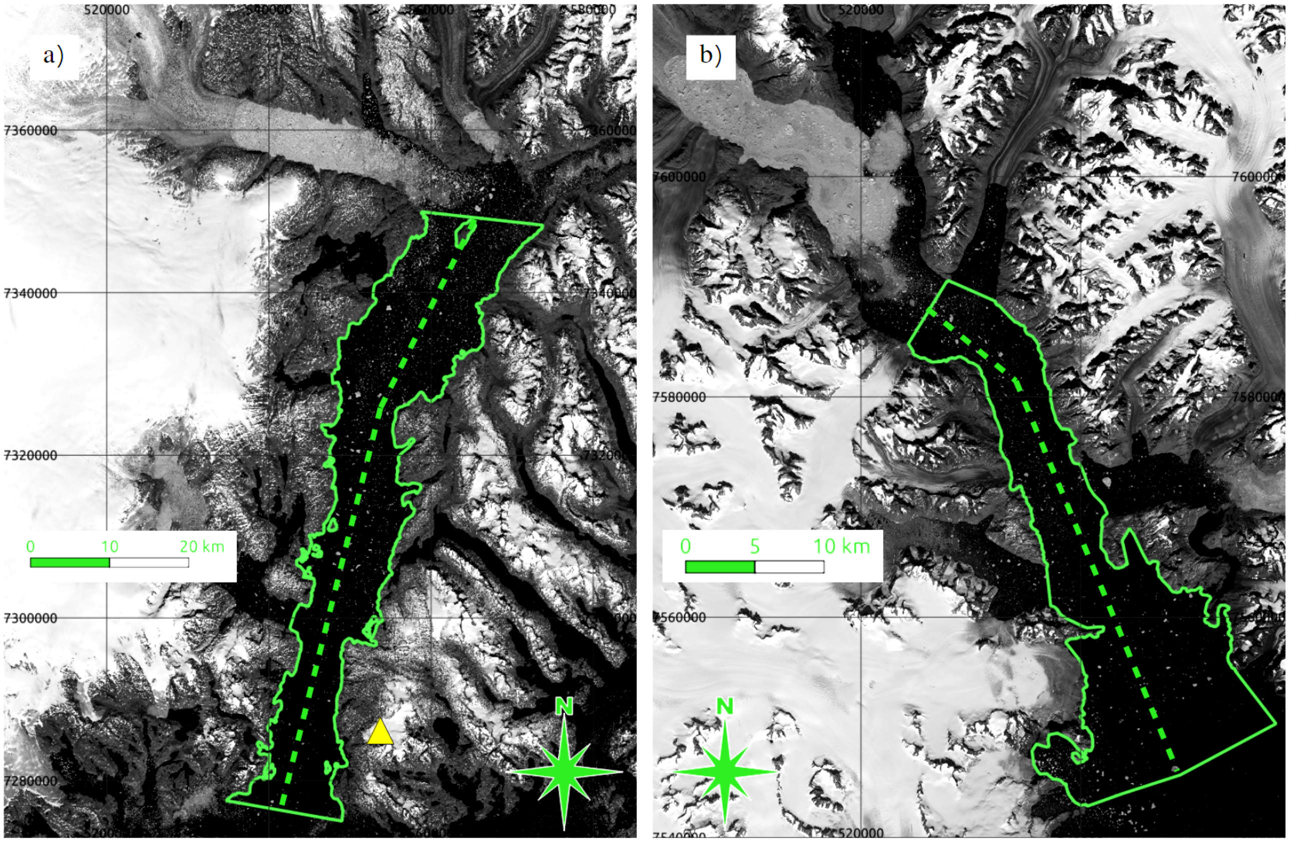

Supplementary Figure 1: Sentinel-2 mosaics for a) Sermilik (June 15, 2019) and b) Kangerlussuaq (July 7, 2019) fjords. Dashed lines within the boundaries of each fjord show the approximate lines along which the iceberg distributions are shown in Figs. 4 and 5 of the main text. Location of PROMICE MIT weather station is shown with a yellow triangle[1].

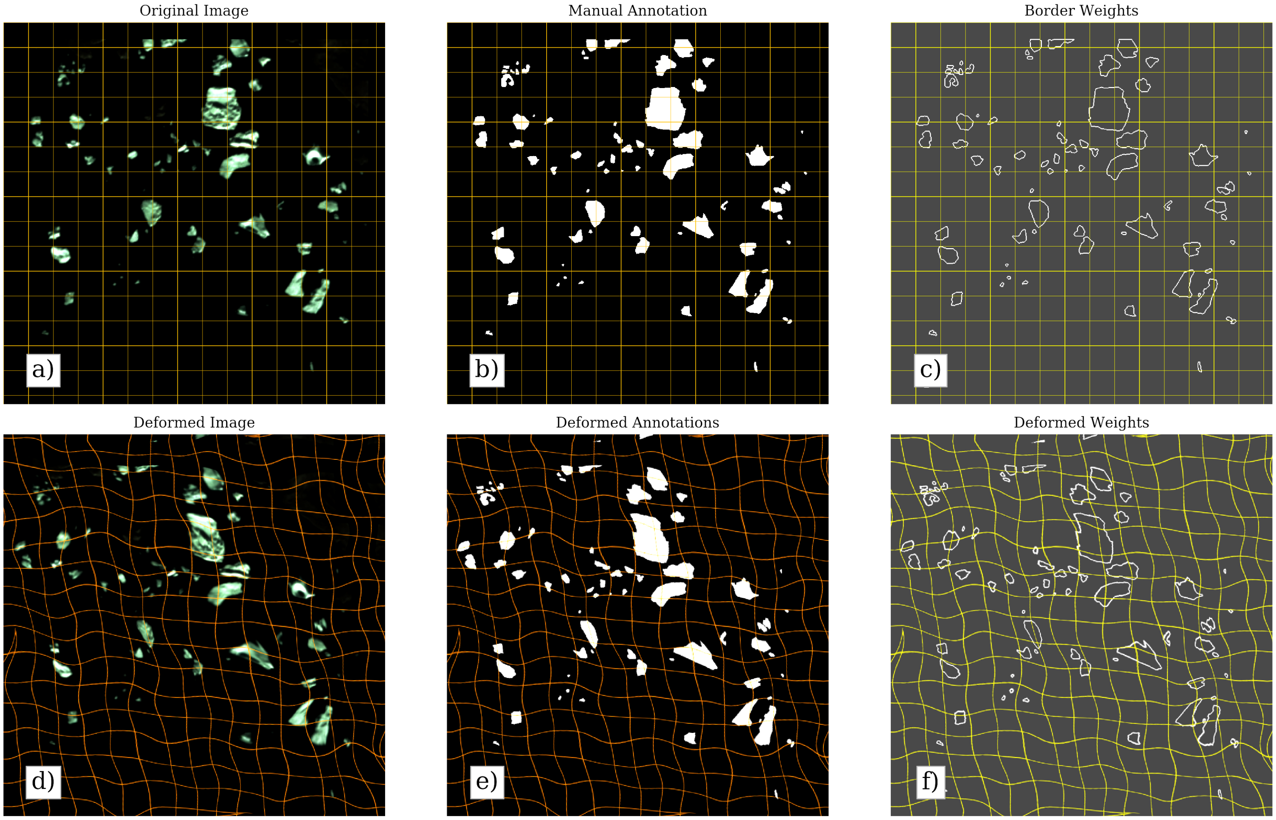

Supplementary Figure 2: Example of a) sub-images, b) manual annotation, c) and weight-map used for calculating the loss function, with their corresponding elastic deformations applied for data augmentation (d-f). In panels c) and f), the white borders have a larger weight map ($\mathcal{W} = 5$) than the rest of the image ($\mathcal{W} = 1$). Image size is 768×768 pixels equivalent to 2304×2304 m.

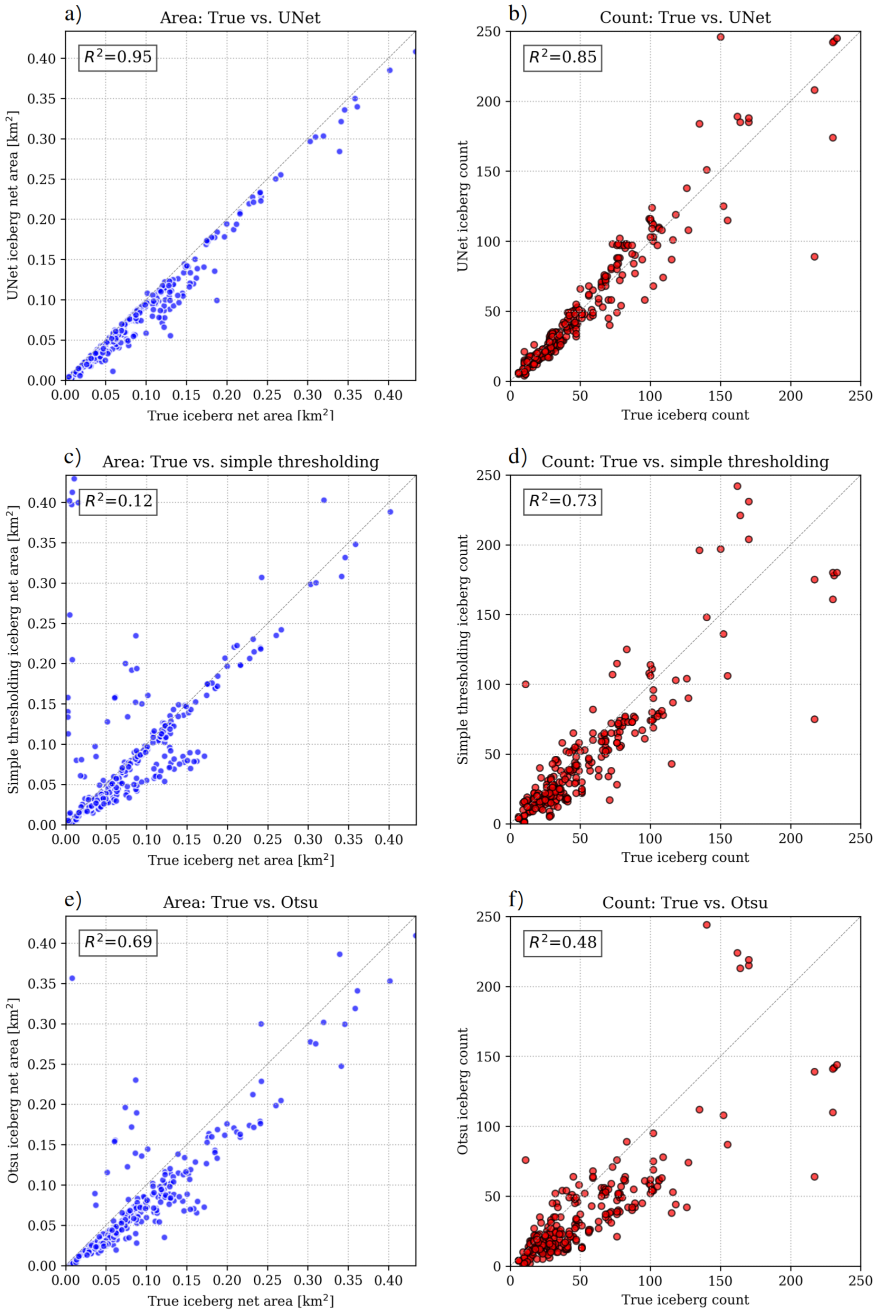

Supplementary Figure 3: Comparing the results of different segmentation methods against manual annotation for total area in each validation image (*left column*) and number of detected icebergs (*right column*) for a,b) UNet, c,d) global thresholding method, and e,f) Otsu thresholding. Each circle represents a validation image. The $x-$axes show the manual annotation results for iceberg area and count, while $y-$axes are same parameters obtained from different segmentation methods.

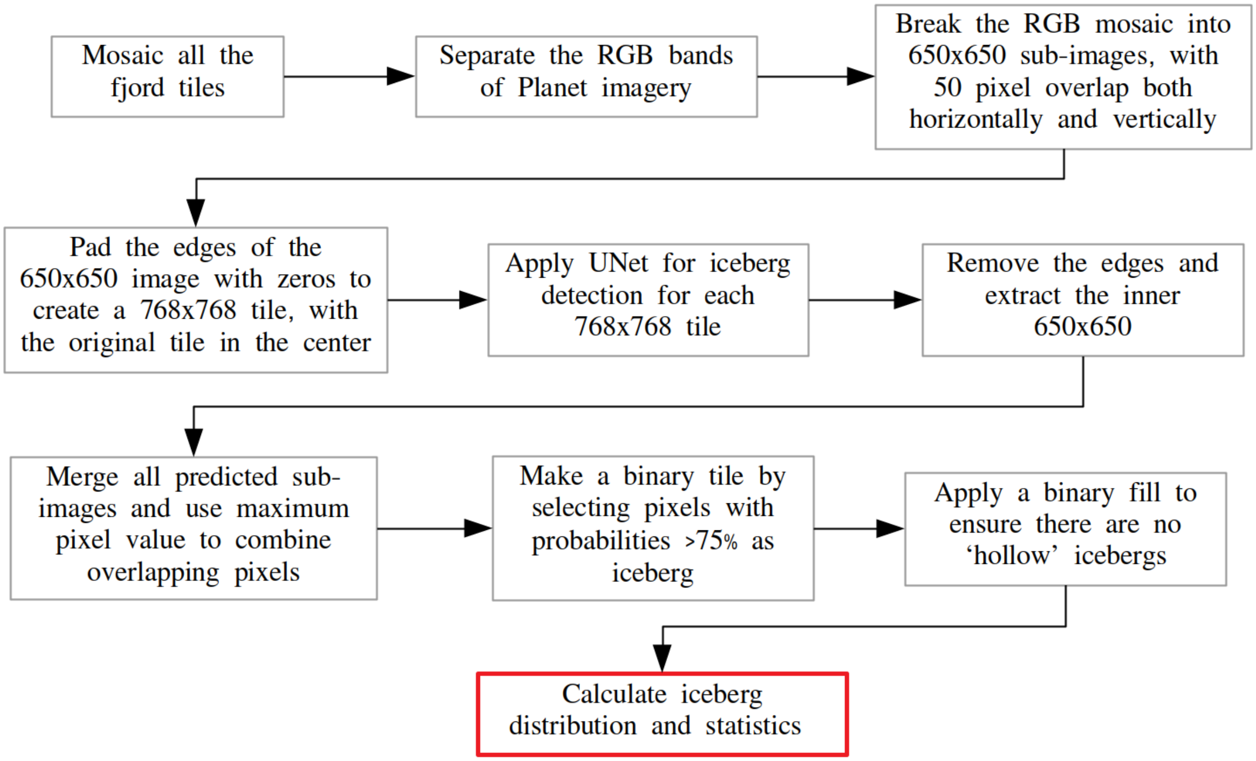

Supplementary Figure 4: Steps for detecting icebergs using UNet on a mosaic of Planet tiles.

Supplementary Tables

Supplementary Table 1: Sermilik Fjord imagery.

imagery	unique IDs
Sentinel-2	L1C_20190615T141011_N0207_R053_T24WWT_20190615T142429
	L1C_20190615T141011_N0207_R053_T24WWU_20190615T142429
Planet	20190615_134220_1032_3B_Analytic
	20190615_134221_1032_3B_Analytic
	20190615_134222_1032_3B_Analytic
	20190615_134223_1032_3B_Analytic
	20190615_134224_1032_3B_Analytic
	20190615_134225_1032_3B_Analytic
	20190615_134226_1032_3B_Analytic
	20190615_134227_1032_3B_Analytic
	20190615_134228_1032_3B_Analytic
	20190615_134229_1032_3B_Analytic
	20190615_134230_1032_3B_Analytic

Supplementary Table 2: Kangerlussuaq Fjord imagery.

imagery	unique IDs
Sentinel-2	L1C_20190707T135739_N0207_R010_T25WER_20190707T141452
	L1C_20190707T135739_N0207_R010_T25WES_20190707T141452
Planet	20190707_120511_39_1063_3B_AnalyticMS
	20190707_120509_31_1063_3B_AnalyticMS
	20190707_120507_23_1063_3B_AnalyticMS
	20190707_120505_15_1063_3B_AnalyticMS

Supplementary Table 3: P-UNet error differentiated by iceberg size in the validation dataset. The mean weighted error $\bar{E}$ ∼12% (calculated using Eq. S2).

**iceberg size range [m^2]**	**mean percentage error**	ΣA **[km^2]**
A < 10^3	18%	3.7
$10^3 \leq$ A < 5×10^3	20%	4.8
$5\times10^3 \leq$ A < 10^4	19%	2.5
$10^4 \leq$ A < 10^5	9%	12.0
$10^5 \leq$ A	2%	3.8

Supplementary Notes, Discussion, Methods

S1 Data

We use PlanetScope data from ©Planet Labs Inc.; each PlanetScope satellite is a CubeSat 3U form factor with an altitude of 400 km for international space station orbit and 475 km for the sun-synchronous orbit with daily repeat time. PlanetScope imagery is provided in a four-band multi-spectral image (red 590-670 nm, green 600-590 nm, blue 445-515 nm, near-infrared 780-860 nm) or three-band natural color (red, green, blue). In order to generate uniform training data, we limit the training data to RGB bands of both 3-band or 4-band imagery. Although we perform an end-to-end training for iceberg detection, choosing RGB bands also facilitates transfer learning of the current trained model to other optical satellite imagery that have similar

spectral ranges for RGB bands. A list of unique IDs for all imagery used in this study is listed in Supplementary Tables 1 and 2.

Planet imagery is provided in individual tiles (a.k.a. scenes) varying from 20 km $\times$ 12 km to 24.6 km $\times$ 16.4 km for international space station orbit and sun-synchronous orbits, respectively, with an average spatial resolution of $\sim$3 m (small variations depend on orbit). We use imagery with less than 10% cloud cover. There are 11 tiles covering Sermilik Fjord on June 15th, 2019, and 4 tiles for Kangerlussuaq Fjord on July 7th, 2019. We mosaic all the tiles (Supplementary Fig. 1) and remove all the marginal fjord walls and rocks. Since the spectral properties of the mélange is substantially different from icebergs in open water, we limit our results to non-mélange regions; segmenting individual icebergs in mélange requires additional labels and training, solely focused on the mélange regions.

S2 Training

S2.1 UNet Architecture

Convolutional Neural Networks (CNN) have been widely used to analyze image contexts[2]. CNNs capabilities have been extended to performing semantic segmentation tasks, where every pixel is assigned to belong to a certain object. Among the most successful implementations of CNNs is the UNet architecture developed by Ref. [3] that was originally used for biomedical imaging studies.

UNet architecture consists of two symmetric branches, namely, a contracting (or down-sampling) branch and an expansive (up-sampling) branch. In the first branch, consecutive unpadded convolution filters are applied to the image with a rectified linear unit (ReLU) activation function. This step is followed by a 2 $\times$ 2 max-pooling operation, and the process is repeated as specified by the number of layers (4 layers in our case). At every layer, the number of extracted features are doubled. Since applying convolution layers and max-pooling operations result in down-sampling of the input image, an up-sampling procedure is needed to obtain the same input image. This task is performed by the second up-sampling branch of UNet; rather than applying max-pooling, an "up-convolution" operation is applied which halves the number of features compared to the previous one. The results of this step are concatenated

to the features from the corresponding layer in the contracting branch of the network. A final 1×1 convolution is applied to map the features from the last layer to the specified labels in the training data. In the end, pixel-wise softmax results in the probability map, that assigns the likelihood of assigning every pixel to each class[3;4].

S2.2 Data Augmentation and Training Set-up

To provide training data for the deep learning algorithm, we break the tiles into sub-images ranging from $\sim 150 \times 150$ to 700×700 pixels. We choose these images from three Greenlandic fjords in east and west Greenland to incorporate the possible differences in PlanetScope images in our training data. We then manually annotate individual icebergs in each sub-image using the Visual Geometry Group annotation tool[5]. Data augmentation is done using elastic deformation and random rotation to increase the training data; the ratio of augmented sub-images using elastic deformation to random rotation is 9 to 1.

Although we take extreme care when annotating the images, some small icebergs may be smaller than the size of the computer cursor and left unlabeled. Therefore, it is possible that our manual annotation does not fully annotate the small icebergs, leading to a slightly higher false negative-rate in manual annotations. In some cases differentiating between a turbulent ocean water and a small iceberg is not possible. We did not perform any pre-processing to sharpen the images or apply any filter to remove possible noise from the images throughout this work; this is another advantage of the present work compared with previous deep learning or generic vision studies for iceberg detection or glacier terminus tracking[6;7;8;9]. However, we note that pre-processing the images can increase the likelihood of applying the trained network on other optical imagery. Providing such a network is beyond the purpose of the present work.

Identifying icebergs in a fjord has two main objectives: 1) identify the net area of icebergs in the fjord for oceanographic purposes, and 2) estimating the frequency-size distribution of icebergs for calving style inferences. The first objective is precisely the task of semantic segmentation. However, the second objective requires identifying individual icebergs with proper delineation of their edges. If two icebergs are in very close proximity of each other, semantic segmentation may predict the total area very accurately, but the frequency-size distribution can be incorrect if the two icebergs are not differentiated properly. While UNet is very power-

ful in semantic segmentation of images, it must be slightly modified for instance segmentation purposes.

Since we aim at identifying icebergs only, the total number of classes are 2 (iceberg, and non-iceberg). Therefore, we define a weighted binary cross-entropy loss function $\mathcal{L}$ for training as

$$\mathcal{L} = -\sum_{i=1}^{n^*} \mathcal{W}_i \big(y_i \, \log(p_i) + (1 - y_i) \, \log(1 - p_i) \big), \tag{S1}$$

where $\mathcal{W}$ is the weight function, y is the binary prediction, p is the probability of assigning a pixel to the iceberg class. The loss function is calculated for each pixel i, and summed over n^* that represents the total number of pixels in an image. This weight function penalizes the borders of each iceberg five times greater than the interiors of an iceberg or the background pixels (see Supplementary Fig. 2).

We train UNet with 4 layers and perform cross-validation by splitting the label data to ~80% training and ~20% validation, and network training is done through 50 epochs with 100 iterations in each epoch. The best model is chosen based on minimizing the loss function $\mathcal{L}$. We minimize the loss function $\mathcal{L}$ using adaptive moment (a.k.a. Adam) optimizer[10] with a learning rate of 5×10^{-5}. Although we did not encounter overfitting during hyperparameter tuning, we include a dropout of 0.75 to ensure the network does not overfit[11].

S2.3 Validation and Error Analysis

An ideal metric for evaluating the performance of the trained model is to compare frequency size distribution of icebergs from manual annotations and model predictions. However, since large-scale test data of an entire fjord does not exist, we propose a different approach for evaluating the algorithm's performance.

We evaluate the prediction error for every sub-image in the test set. The first metric to consider is the total number of iceberg pixels identified. If an annotated sub-image has n icebergs with sizes $\{A_1, A_2, ..., A_n\}$, then the total number of iceberg-labeled pixels is $A_l = \sum_{i=1}^{n} A_i$. Similarly, the predicted sub-image may have m icebergs with sizes $\{\hat{A}_1, \hat{A}_2, ..., \hat{A}_m\}$, with the sum of pixels as $\hat{A}_p = \sum_{i=1}^{m} \hat{A}_i$. In an ideal case, $m = n$ and $A_l = \hat{A}_p$. Therefore, we choose A_l vs. $\hat{A}_p$ and $n - m$ for every sub-image as metrics for evaluating the performance of our model (Supplementary Fig. 3).

The R^2 values reported in Supplementary Fig. 3 holistically demonstrate the success of our trained network, however, they do not necessarily hold for all iceberg sizes. Segmenting small icebergs is significantly more challenging than large icebergs. Therefore, we report the segmentation error based on different iceberg sizes in validation set (Supplementary Table 3). For icebergs greater than 10^5 m^2, the segmented iceberg area via P-UNet is <2%. The error, however, increases with for icebergs smaller than 10^4 m^2 to an average of ~19%. We calculate the average error weighted by iceberg area in validation set as

$$\bar{E} = \frac{\sum E_i A_i}{\sum A_i}, \tag{S2}$$

where i denotes the iceberg size range in Supplementary Table 3. This results in an average of error of $\bar{E}$ ~12% for P-UNet.

Note that Ref. [7] reports 12-18% error for icebergs larger than 10^5 m^2 detected using S-TOA, significantly larger than our estimates for same size icebergs. We expect that the error will be much higher for icebergs smaller than 10^5 m^2, however, we use 18% error for all icebergs sizes detected with S-TOA.

Although the metrics provided above are useful for the specific goal of frequency-size distribution, they do not provide grounds for comparison against future studies. Hence we present additional model evaluation metrics that allow such comparisons. In the majority of images that are used for training, non-iceberg regions form the majority of pixels. Therefore, the classification problem we present is highly imbalanced and F1 score is an appropriate classification metric. F1 score is a combination of precision and recall defined as

$$\text{precision} = \frac{TP}{TP + FP}, \qquad \text{recall} = \frac{TP}{TP + FN}, \tag{S3}$$

where (T, F) refers to (true, false), and (P, N) refers to (positive, negative) detections. F1 score can then be calculated as

$$\text{F1} = \frac{2 \times \text{precision} \times \text{recall}}{\text{precision} + \text{recall}}. \tag{S4}$$

The closer F1 score is to 1, the better the segmentation model is. Our model results in $TP = 0.814$, $TN = 0.996$, $FP = 0.0038$, and $FN = 0.186$, which lead to F1=0.896. Our trained model is robust to false positives ($FP = 0.0038$) and therefore is more 'conservative'. This leads to an overall underestimation of iceberg areas using P-UNet (as also seen in Supplementary Fig.

3a), while S-TOA mostly overestimates iceberg area and has a large false positive rate (see SI of Ref. [7] for details).

S3 Iceberg detection workflow

Because the size of the mosaiced Planet tiles that cover an entire fjord is very large, it cannot immediately be processed by regular graphical processing units (GPUs). Therefore, we divide the mosaic into several smaller tiles. Iceberg detection using UNet is applied to each tile and the final results are obtained by combining the tiles back together. Steps for performing these steps are shown in Supplementary Fig. 4.

S4 Iceberg melt rate estimates

The three dominant iceberg melting processes are formulated as

$$M_e = a_1|\vec{v}_a|^{1/2} + a_2|\vec{v}_a|, \tag{S5}$$

$$M_v = b_1 T_w + b_2 {T_w}^2, \tag{S6}$$

$$M_b = c|\vec{v}_w - \vec{v}_i|^{4/5}(T_w - T_i)\ L^{-1/5} \tag{S7}$$

where $a_1 = 8.7\times10^{-6}$ m$^{1/2}$s$^{-1/2}$, $a_2 = 5.8\times10^{-7}$, $b_1 = 8.8\times10^{-8}$ m s^{-1} °C^{-1}, $b_2 = 1.5\times10^{-8}$ m s^{-1} °C^{-2}, and $c = 6.7\times10^{-6}$ m$^{-2/5}$s$^{-1/5\circ}$C^{-1}. Surface air velocity, iceberg velocity, and surface water velocity are represented by $\vec{v}_a$, $\vec{v}_i$, and $\vec{v}_w$, respectively, and T_w denotes the sea surface temperature (SST) at 4°C. Since basal melt is ignored in our analysis, the derived formulation is independent of parameters in M_b. Equations (S5-S7) are valid when $|\vec{v}_a| \gg |\vec{v}_i|$[12].

The time-dependent freshwater production from iceberg melt is derived by using empirical relations of Ref. [13], where width, W, and keel depth,D, are expressed as a function of length, L, as $W = \frac{L}{1.62}$ and $D = 2.91\times L^{0.71}$. These empirical relationships are obtained based on a number of detail iceberg geometric measurements at depth, however, it is possible that they don't represent the entire spectrum of iceberg geometries and shapes. Employing these geometric approximations results in an explicit relationship for iceberg volume, V, as a function of L as

$$V = 1.8\times L^{2.71}. \tag{S8}$$

By definition, we know $\frac{dL}{dt} = -M_e - M_v$. Integrating this equation to derive a time dependent length reads

$$L(t) = -(M_e + M_v)t + L_0, \tag{S9}$$

with t as time (in seconds) and L_0 the initial iceberg length. Equation (S9) can be readily applied in the volume-length relationship (eq. S8) to express the iceberg volume as a function of time. Hence,

$$V(t) = 1.8 \times \left[-(M_e + M_v)t + L_0\right]^{2.71}. \tag{S10}$$

Note that Eq. (S10) is derived by ignoring the basal melt rate, M_b. Detailed melt rate calculations of Ref. [14] show that wave erosion, M_e, is the dominant parameter for iceberg melt rate and is an order of magnitude greater than M_b, especially during summer. Therefore, since the focus of our analysis is small icebergs, the overall impact of ignoring basal melt rate is not significant. For estimating the life span of icebergs we use $\vec{v}_a$ as the mean recorded wind velocity during June-July 2019 from PROMICE MIT weather station [1] (see Fig. S1).

By multiplying equation (S10) by ice density of $\rho_{\text{ice}} = 917$ kg m^{-3}, the time dependent mass of the iceberg can be determined. Therefore, $M(t) = 1.8 \times \rho_{\text{ice}} \left[-(M_e + M_v)t + L_0\right]^{2.71}$. The life span of an iceberg ($\mathcal{T}^*$) can be derived by setting $V(t) = M(t) = 0$, so,

$$\mathcal{T}^* = \frac{L_0}{M_e + M_v}. \tag{S11}$$

References

[1] Dirk van As, Robert S Fausto, Andreas P Ahlstrøm, Signe B Andersen, Morten L Andersen, Michele Citterio, Karen Edelvang, P Gravesen, Horst Machguth, Faezeh M Nick, et al. Programme for monitoring of the Greenland Ice Sheet (PROMICE): first temperature and ablation records. *Geological Survey of Denmark and Greenland Bulletin*, 23:73–76, 2011.

[2] Alex Krizhevsky, Ilya Sutskever, and Geoffrey E Hinton. Imagenet classification with deep convolutional neural networks. In *Advances in Neural Information Processing Systems*, pages 1097–1105, 2012.

[3] Olaf Ronneberger, Philipp Fischer, and Thomas Brox. U-net: Convolutional networks for

biomedical image segmentation. In *International Conference on Medical Image Computing and Computer-assisted Intervention*, pages 234–241. Springer, 2015.

[4] Joel Akeret, Chihway Chang, Aurelien Lucchi, and Alexandre Refregier. Radio frequency interference mitigation using deep convolutional neural networks. *Astronomy and Computing*, 18:35–39, 2017.

[5] Abhishek Dutta and Andrew Zisserman. The VIA annotation software for Images, Audio and Video. *arXiv preprint arXiv:1904.10699*, 2019.

[6] Daniel Sulak, David A Sutherland, Ellyn M Enderlin, Leigh A Stearns, and Gordon S Hamilton. Iceberg properties and distributions in three Greenlandic fjords using satellite imagery. *Annals of Glaciology*, 58(74):92–106, 2017.

[7] Alexis Moyer, David Sutherland, Peter Nienow, and Andrew Sole. Seasonal variations in iceberg freshwater flux in sermilik fjord, southeast greenland from sentinel-2 imagery. *Geophysical Research Letters*, 46(15):8903–8912, 2019.

[8] Yara Mohajerani, Michael Wood, Isabella Velicogna, and Eric Rignot. Detection of glacier calving margins with convolutional neural networks: a case study. *Remote Sensing*, 11(1):74, 2019.

[9] Celia A Baumhoer, Andreas J Dietz, Christoph Kneisel, and Claudia Kuenzer. Automated Extraction of Antarctic Glacier and Ice Shelf Fronts from Sentinel-1 Imagery Using Deep Learning. *Remote Sensing*, 11(21):2529, 2019.

[10] Diederik P Kingma and Jimmy Ba. Adam: A method for stochastic optimization. *arXiv preprint arXiv:1412.6980*, 2014.

[11] Nitish Srivastava, Geoffrey Hinton, Alex Krizhevsky, Ilya Sutskever, and Ruslan Salakhutdinov. Dropout: a simple way to prevent neural networks from overfitting. *The Journal of Machine Learning Research*, 15(1):1929–1958, 2014.

[12] Torge Martin and Alistair Adcroft. Parameterizing the fresh-water flux from land ice to ocean with interactive icebergs in a coupled climate model. *Ocean Modelling*, 34(3-4):111–124, 2010.

[13] Anne Barker, Mohamed Sayed, T Carrieres, et al. Determination of iceberg draft, mass and cross-sectional areas. In *The Fourteenth International Offshore and Polar Engineering Conference*. International Society of Offshore and Polar Engineers, 2004.

[14] Twila Moon, David Sutherland, Dustin Carroll, Denis Felikson, Laura Kehrl, and Fiamma Straneo. Subsurface iceberg melt key to Greenland fjord freshwater budget. *Nature Geoscience*, 11(1):49, 2018.
